# The percutaneous coronary intervention prior to transcatheter aortic valve implantation (ACTIVATION) trial: study protocol for a randomized controlled trial

**DOI:** 10.1186/1745-6215-15-300

**Published:** 2014-07-24

**Authors:** Muhammed Zeeshan Khawaja, Duolao Wang, Stuart Pocock, Simon Robert Redwood, Martyn Rhys Thomas

**Affiliations:** The Rayne Institute, Cardiovascular Division, King’s College London, BHF Centre of Excellence, St Thomas’ Hospital, Westminster Bridge Road, London, SE1 7EH UK; Department of Cardiology, Guy’s & St Thomas’ NHS Foundation Trust, Westminster Bridge Road, London, SE1 7EH UK; Department of Clinical Sciences, Liverpool School of Tropical Medicine, Pembroke Pl, Liverpool, Merseyside L3 5QA UK; Department of Medical Statistics, London School of Hygiene and Tropical Medicine, Keppel St, Bloomsbury, London WC1E 7HT UK

**Keywords:** Transcatheter aortic valve implantation, Percutaneous coronary intervention, Aortic stenosis, Coronary

## Abstract

**Background:**

Current guidelines recommend treatment of significant coronary artery disease by concomitant coronary artery bypass grafting (CABG) in patients undergoing surgical aortic valve replacement. However there is no consensus as to how best to treat coronary disease in high-risk patients requiring transcatheter aortic valve implantation (TAVI).

**Methods/Design:**

The percutaneous coronary intervention prior to transcatheter aortic valve implantation (ACTIVATION) trial is a randomized, controlled open-label trial of 310 patients randomized to treatment of significant coronary artery disease by percutaneous coronary intervention (PCI - test arm) or no PCI (control arm). Significant coronary disease is defined as ≥1 lesion of ≥70% severity in a major epicardial vessel or 50% in a vein graft or protected left main stem lesion. The trial tests the hypothesis that the strategy of performing pre-TAVI PCI is non-inferior to not treating such coronary stenoses with PCI prior to TAVI, with a composite primary outcome of 12-month mortality and rehospitalization. Secondary outcomes include efficacy end-points such as 30-day mortality, safety endpoints including bleeding, burden of symptoms, and quality of life (assessed using the Seattle Angina Questionnaire and the Kansas City Cardiomyopathy Questionnaire).

In conclusion, we hope that using a definition of coronary artery disease severity closer to that used in everyday practice by interventional cardiologists - rather than the 50% severity used in surgical guidelines - will provide robust evidence to direct guidelines regarding TAVI therapy and improve its safety and efficacy profile of this developing technique.

**Trial registration:**

ISRCTN75836930, http://www.controlled-trials.com/ISRCTN75836930 (registered 19 November 2011).

## Background

Transcatheter aortic valve implantation (TAVI) has moved into the cardiology mainstream with rapid acceptance of this new technology since the first implant in 2002 [1]. The two devices with the largest experiences are the self-expanding CoreValve Revalving™ system (Medtronic CoreValve, Luxembourg) and the balloon expandable Edwards Sapien XT valve (Edwards Lifesciences, Irvine, CA). Both are employed in patients whose peri-operative risk is deemed too high for surgical aortic valve replacement (sAVR). Coronary artery disease has a high prevalence in these patients [2–5] and shares many of the same causative factors [6]. While the matrices for these devices state that significant coronary artery disease (CAD) should preferably be treated prior to implantation, current practice is frequently not to do so. Indeed, successful PCI has been demonstrated post-implantation [7–9], as both technologies allow access to the coronary ostia. The presence of concomitant CAD has been associated with adverse procedural outcomes in sAVR [10, 11] and now also in TAVI [5]. In this higher risk cohort, with a mean age in excess of the ‘normal’ population with chronic stable angina, we must carefully evaluate any coronary artery lesion’s significance in terms of the possibly greater risks of PCI and the impact on the planned valvular intervention.

### Considerations in pre-transcatheter aortic valve implantation percutaneous coronary intervention

Among the possible advantages of revascularization prior to TAVI may be a protective effect against the ischemic burden of the procedure, including as it does periods of hypotension. The absence of contractile reserve is associated with increased mortality after sAVR [12], and significant stenoses not intervened upon could contribute to this. Surgical revascularization for multi-vessel coronary artery disease has been found to be an independent factor predictive of improvement of left ventricular ejection fraction (LVEF) after sAVR [13], and similar benefits for revascularization by PCI may exist. Improving coronary flow in symptomatic patients with significant flow-limiting stenoses may maximize this beyond the valvular intervention. Wave intensity analysis of coronary flow has demonstrated marked reduction in the diastolic suction wave in aortic stenosis (the dominant wave in coronary perfusion [14]), which significant stenosis may impair further [15].

The risks of PCI are well described: death, myocardial infarction, coronary artery bypass grafting (CABG), stroke, vascular access complications, renal insufficiency, allergy and stroke/TIA [16, 17]. PCI is also associated with an incidence of stent thrombosis of up to 1% with significant mortality [18, 19]. The presence of severe aortic stenosis could have a detrimental effect upon the ability to withstand these. Indeed, the hypotension experienced by patients during the TAVI procedure (especially during rapid right ventricular pacing) may actually increase the risk of stent thrombosis. There has been increasing recognition recently of the adverse outcomes associated with major bleeding post-PCI [20, 21]. Current opinion is to continue aspirin for life and clopidogrel for one month after bare metal stenting and 12 months after insertion of a drug-eluting stent. This would possibly impact upon bleeding complications in transfemoral, subclavian and transaortic TAVI and would certainly be of concern in patients undergoing TAVI via the transapical approach, given the direct myocardial access required. Angiography in the 24 hours, or even the 5 days, prior to sAVR has been shown to be associated with acute kidney injury post-operatively [22]. These risks may also exist if PCI and TAVI are inadequately separated or combined in a hybrid procedure. Finally, patients would require two admissions with attendant implications to cost and risk. This latter consideration might be offset against the possible reduction in length of stay in the post-TAVI period.

### Why do we need a randomized trial?

The data on coronary artery disease in TAVI and PCI in this context is certainly provocative, but there are several major limitations, given that the studies are all nonrandomized, registry-type with relatively small sample sizes.

The lack of data on pre-procedural revascularization has led to some variability between centers on the level of coronary interventions undertaken prior to implantation. A retrospective analysis by Masson *et al*. stratified TAVI patients according to myocardium at risk due to coronary artery disease, finding no difference in 30-day or 1-year mortality between the groups [4]. However the small group sizes involved would have required a mortality difference between even the two most extreme groups at 30 days of greater than one third to gain significance, limiting our use of this data. The access approach used was not included in their risk model, an important omission given the known differences in risk profiles between, for example, TF-TAVI and TA-TAVI cohorts. In the German TAVI registry, CAD was defined as previous revascularization (either PCI or CABG) OR a stenosis of ≥50% and was noted in 62% of 1,382 patients. These patients had a greater in-hospital mortality (10.0 versus 5.5%, *P* <0.01), required more frequent cardio-pulmonary resuscitation (7.8 versus 3.5%, *P* <0.01) and suffered greater 30-day mortality (log rank *P* = 0.041). But there were significant differences in the group demographics: more frequent peripheral vascular disease, lower left ventricular (LV) ejection fraction and higher logistic EuroScores in patients with CAD [23]. The Italian CoreValve registry noted that in patients with critical ostial disease, myocardial infarction (MI) within 12 months of TAVI was greater in those patients who were not revascularized prior to TAVI [24]. A recent meta-analysis of the effects of CAD upon outcome in the midterm showed no effect; but again, of seven included studies, five defined CAD as previous revascularization, and the remaining two used 50% stenosis severity [25].

The presence of pre-existing treated coronary artery disease has been identified as an adverse risk factor for procedural and long term outcome [5]. With regards to the effects of pre-TAVI PCI, Masson and colleagues performed a sub-analysis of just 15 patients in whom PCI was performed at the discretion of the individual cardiologist: the resultant selection bias in turn clouds the interpretation of any findings. The mortality in the overall group one-year after TAVI was reported as 77.9% and was found to be 80% in those with pre-emptive PCI - though no comparison between the two cohorts was performed [4]. Abdel Wahab *et al*. reported that 55 patients who underwent PCI prior to TAVI showed no difference in adverse events at either 30 days (2% versus 6%; *P* = 0.27) or 6 months (9% versus 14%; *P* = 0.42) when compared to a group undergoing isolated TAVI [26]. The feasibility of PCI for CAD in patients with severe AS has been demonstrated by Goel *et al*. in a cohort of 258 patients over a 10-year period with a favorable comparison with propensity matched patients without the aortic disease [27].

There has been a rapid, global expansion in the use of TAVI to treat aortic stenosis in patients who are not candidates for sAVR. The efficacy of this technique has been successfully demonstrated in randomized, controlled trials [28, 29] and in large registries [30, 31], and there is now a need to improve patient safety and outcome, including how to manage patients with concomitant CAD. Given the paucity of data on the issue and variation in practice and recommendations we feel a randomized controlled trial (RCT) is essential for safe evidence-based practice. ACTIVATION is the first randomized controlled trial of elective PCI prior to TAVI. Its findings will help define the optimum revascularization strategy in this procedure and help create evidence-based guidelines on this controversial issue.

## Methods/Design

### Hypothesis, design and end points

ACTIVATION is a prospective, randomized, open-label trial that aims to test the hypothesis that revascularization of significant coronary artery disease by PCI prior to TAVI (Test arm) will result in a rate of mortality and re-hospitalization at 12 months that is non-inferior to TAVI without such revascularization (control). Because the control patients in this study are receiving a CE/FDA approved valve replacement, a trial arm comparison would not be likely to demonstrate superiority in either direction.

The trial has an intentional non-inferiority design. The safety of TAVI with revascularization performed as deemed necessary by the Heart Team has been demonstrated in previous trials and registries [29, 30, 32]. From the available data detailed in the statistical design section, our hypothesis is that the no PCI strategy will have comparable effectiveness to the PCI (the active control) strategy, hopefully helping the cardiology community decide whether the extra procedure of pre-TAVI PCI can be safely avoided. From an ethical standpoint, a sham procedure in the no PCI arm could not be countenanced. Given the complexities of the TAVI procedure and the recommended Heart Team approach, we could not blind operators as to whether patients had undergone revascularization or not. To minimize bias towards the null, we have robust inclusion/exclusion criteria and clear end-point definitions and boundaries. Any deviation from the protocol in delivery of strategy will be carefully recorded, including any concomitant therapies.

### Patient population

Patient eligibility will be confirmed by the study investigators according to the inclusion and exclusion criteria in Table 1.

**Table 1:** **Eligibility criteria for the percutaneous coronary intervention prior to transcatheter aortic valve implantation (ACTIVATION) trial**

Inclusion criteria	
	Definition
• Patients ≥18 years of age	
• Severe aortic stenosis, as defined by	• Peak transvalvular gradient of ≥40 mmHg on transthoracic echocardiography (TTE), transesophageal echocardiography (TOE) or dobutamine stress echocardiography (DSE)
	• Aortic valve area of <1.0 cm^2^
• Symptoms suggestive of aortic stenosis	Dyspnea, syncope *etc.* but not pre-dominantly angina
• Deemed of prohibitive risk for open aortic valve replacement by a TAVI multidisciplinary team (MDT) and accepted for TAVI by said TAVI MDT.	
Significant coronary artery disease	• ≥1 stenosis of ≥70% in a major epicardial artery deemed suitable for PCI (≥50% if protected left main stem or vein graft)
• Undergoing TAVI via any accepted approach	Transfemoral, transapical, subclavian or transaortic
• Written informed consent	
**Exclusion Criteria**	Definition
• Recent acute coronary syndrome	Within 30 days of randomization
• Unprotected left main stem disease	
• Significant angina	CCS class ≥3
• Pregnancy	
• Active internal bleeding (except menstruation)	
• Allergy to heparin or GPIIb/IIIa inhibitors	
• Thrombocytopenia cells/mm^3^)	Platelet count <100,000
• Patients who have previously been enrolled in this study	
• Patients who are currently enrolled in any other study where involvement in ACTIVATION would involve deviation from either protocol	

TAVI, transcatheter aortic valve implantation; CCS, Canadian Cardiac Society; GpIIb/IIIa, glycoprotein IIb/IIIa.

### Primary endpoint

The primary endpoint is a composite of mortality and rehospitalization at 12 months since randomization. Mortality is defined as death due to any cause, the exact nature and date of which will be recorded. All deaths will be considered cardiovascular-related unless there is documentation to the contrary.

### Secondary endpoints

These consist of:

i) Mortality at 30 days and 12 months post-TAVI
ii) Major adverse cardiovascular or cerebrovascular event (MACCE) at 30 days and 12 months
iii) Acute myocardial infarction (MI) at 30 days and 12 months
iv) Stroke at 30 days and 12 months
v) Repeat revascularization by either PCI or CABG
vi) Hospitalization for heart failure at 30 days and 12 months
vii) Procedural complications (ventricular fibrillation (VF) or ventricular tachycardia (VT) requiring cardioversion
viii) Requirement of cardiopulmonary bypass
ix) Cardiopulmonary arrest requiring cardiopulmonary resuscitation (CPR) and/or assisted ventilatory support
x) Procedural success
xi) Bleeding complications
xii) Access site complications
xiii) Cerebrovascular events
xiv) Acute kidney injury
xv) Duration of hospital stay
xvi) Quality of life
xvii) Anginal burden
xviii) 6-minute walk test

The definitions are in accordance with the Valve Academic Research Consortium (VARC) guidelines and its update and are specified in Table 2 [33].

**Table 2:** **Endpoint definitions**

End-point	Definition
**Mortality**	All-cause death
**Cardiovascular mortality**	All deaths unless otherwise clearly documented to be related to another cause (for example, trauma, cancer, suicide *etcetera*)
**Myocardial infarction (MI)**	Peri-procedural MI related to TAVI (≤72 h after the TAVI)
	Spontaneous MI (>72 h after TAVI)
	MI related to PCI (type 4a)
	MI related to PCI due to stent thrombosis (type 4b)
**Cerebrovascular event**	Stroke
	Transient ischemic attack
**Bleeding complication**	Life-threatening or disabling
	Major
	Minor
**Access site complication**	Major
	Minor
**Acute kidney injury** As per AKIN classification in VARC2 [34]	Stage 1
	Stage 2
	Stage 3
**Major adverse cardiac and cerebrovascular events (MACCE)**	Combined cardiovascular mortality, MI, stroke and further revascularization

TAVI, transcatheter aortic valve implantation; PCI, percutaneous coronary intervention, AKIN, acute kidney injury network; VARC2, Valve Academic Research Consortium second guidelines [34].

### Ethics

Ethics review was approved by United Kingdom National Research Ethics Committee (London Dulwich) (11/LO/1596).

### Randomization

**Figure 1:**
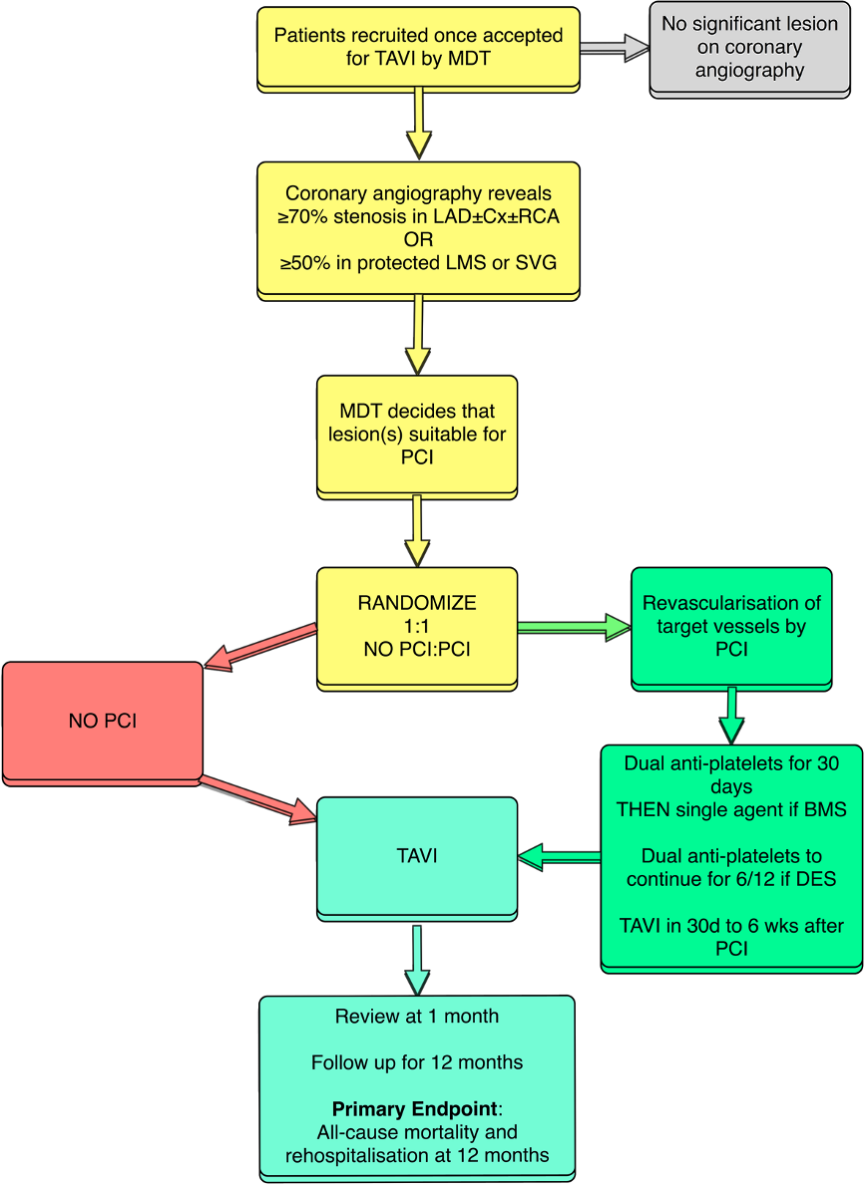
**Flow chart.** (TAVI, transcatheter aortic valve implantation; MDT, multidisciplinary team; LAD, left anterior descending artery; Cx, circumflex artery; RCA, right coronary artery; LMS, left main stem; SVG, saphenous vein graft; PCI, percutenaous coronary intervention; BMS, bare metal stent; DES, drug eluting stent).

Randomization will take place once the patient has provided written, informed consent. This will be performed via an online portal. Randomization of the treatment assignment is based on the block method using randomly varying block sizes and will be stratified by center. Once the patient is randomized s/he will be monitored for the duration of the follow-up irrespective of subsequent clinical management. The intended timeframe for PCI should be stated at randomization. After PCI the patient should receive dual anti-platelet therapy for 30 days - one of which can then stop if the patient receives a bare-metal stent (BMS). All PCI patients should undergo TAVI within the 2 weeks after this 30-day post-PCI time point. Any clinical events which occur in the test arm during this period will be attributed to the percutaneous revascularization on an intention-to-treat-basis. The flow diagram in Figure 1 illustrates the recruitment and study process.

### Trial conduct

ACTIVATION will be conducted at up to 20 hospitals in Europe providing TAVI using the Edwards system. Trial administration and data management will be carried out by an independent clinical trial organization, the European Cardiovascular Research Center, Massy, France. The trial is registered at http://www.controlled-trials.com (ISRCTN75836930).

### Procedural details

#### Percutaneous coronary intervention

The percutaneous coronary intervention in the test arm should be performed according to current local best practice. The use of the OMEGA bare metal stent (Boston Scientific Inc, Natick, MA, USA) or PROMUS family of drug-eluting stents (DES; Boston Scientific Inc, Natick, MA, USA) is mandatory. It is strongly recommended that patients should be pre-treated with aspirin 300 mg and either clopidgrel 600 mg, prasugrel 60 mg, or ticagrelor 180 mg as per local guidelines. Patients should also receive appropriate anticoagulant therapy during the PCI. The route of access (radial or femoral), use of further interventional techniques (for example, intravascular ultrasound, rotablation, laser atherectomy *etcetera*) and PCI equipment are at the discretion of the operator. Post-procedure aspirin should be continued at a maintenance of 75 mg o.d.. Patients should receive either clopidogrel 75 mg o.d., Prasugrel 10 mg o.d. or Ticagrelor 90 mg b.d. for 30 days after PCI for BMS or 6 months for DES as per local guidelines.

#### Transcatheter aortic valve implantation

Patients should be accepted for TAVI by a multidisciplinary team including both an interventional cardiologist and a cardiothoracic surgeon. The procedure should use the balloon-expandable Edwards SAPIEN XT prosthesis (Edwards Lifesciences, Irvine, CA, USA). The choices of prosthesis size and access route (transfemoral, transapical or transaortic) are to be left to the discretion of the operating team. Equally the intraprocedural details (local versus general anesthetic, perioperative imaging *etcetera*) should proceed as per usual practice at the enrolling center. Both aspirin and clopidogrel should be continued for at least 6 months after TAVI implantation with lifelong single anti-platelet therapy thereafter.

#### Data collection and monitoring

Data will be collected at baseline from enrolled patients including demographics, past medical history, previous cardiac investigations and current medication. Patients will undergo transthoracic ± transesophageal echocardiography at baseline, post-procedure, 1 month and 12 months. Twelve-lead electrocardiograms will be obtained at the same time points, with additional traces in the test arm pre- and post-PCI and in either arm after any clinical event thought to represent myocardial infarction and before/after any subsequent revascularization procedure. In addition to routine blood tests, a full blood count will be taken pre- and post-PCI.

Prospective monitoring of adverse and clinical events starts at randomization and continues until 12 months. Patients should be followed up to hospital discharge following PCI. All MACCE will be documented by the research coordinator using the clinical event data form. The same applies for the TAVI procedure. Patients will be followed up at 30 days and 12 months for anginal burden using the Seattle Angina Questionnaire and other end-points. Mortality data will be obtained from the Office of National Statistics in England and Wales, the General Register Office in Scotland and from the appropriate authority in other countries.

All MACCE and other serious adverse events will be recorded in the electronic Case Record Form and reported to the coordinating center within 3 working days of first identification. On receipt of notification of any trial adverse or clinical event, the coordinating center will request additional details, specific to the nature of the event. These episodes will be carefully monitored by the trial coordinator and will form part of the information provided at regular intervals to the Clinical Events and Data Monitoring Committees. The study site will also notify their ethics committee and institutional clinical risk management team according to their local policy. The Clinical Events Committee will be blinded to treatment and will consider each MACCE or adverse event reported and ratify occurrence of an endpoint according to the study definitions (according to a majority).

### Statistical consideration

#### Sample size determination

No randomized trials have been performed of elective PCI versus no PCI in patients undergoing TAVI. In a recent nonrandomized registry, Masson *et al*. reported a 1-year mortality of 20% in 15 patients who underwent PCI prior to TAVI - similar to those who did not at 22.1% [4] - despite the presence of existing coronary disease having been shown to increase mortality in patients undergoing TAVI from 18.4 to 35.7% [5].

With the postulation that improved coronary perfusion may protect against some of the ischemic/hypotensive procedural complications of the TAVI procedure, an improvement in rate of mortality seems likely. PCI may lead to restenosis of the stent, requiring rehospitalization in 5% of patients over 12 months. The rate of rehospitalization with stable angina, acute coronary syndrome or ischemia-related heart failure requiring treatment, including possible PCI, is thought to be in the region of 15% in all patients with CAD. The feasibility assumptions for 12-month mortality are 30% in the control (TAVI only) arm and 20% in the test (TAVI + PCI) arm, with similar rates of hospitalization at 15% in both. Based on the above assumptions, we performed sample size simulations and the simulated results show that with a non-inferiority margin of 7.5% and a loss to follow up of 5%, the sample size of 310 patients will have at least 88.8% power to demonstrate non-inferiority of TAVR following PCI compared with TAVR without PIC in the primary endpoint.

### Statistical analysis

For the primary endpoint analysis, cumulative event rate will be calculated using the Kaplan-Meier estimates, and the Greenwood standard errors for these estimates. A 95% one-sided upper confidence limit will be computed for the difference in the primary composite outcome of mortality of rehospitalization at 12 months between arms (Test - Control). The Test arm (PCI) will be judged not inferior to the Control arm (no PCI) if the upper confidence limit is less than 7.5% - the predetermined non-inferiority margin. This was chosen based upon expert opinion and precedent (PARTNER cohort A [29]). In addition to formal analysis of non-inferiority endpoints, the Kaplan-Meier curves will be presented and compared by log rank test by treatment group and hazard ratio and its 95% confidence interval will be calculated using Cox regression model. Also, the combination of the all cause mortality and time to first recurrent hospitalization will be analyzed using the unmatched win ratio approach [35].

For secondary time-to-event outcomes, event rates at 30 and 365 days will be calculated using the Kaplan-Meier method and compared by log rank test. Their hazard ratios will be estimated using Cox regression model. Outcomes with repeated measurements will be analyzed using generalized linear mixed models. Continuous variables will be summarized using number of observations, median (interquartile range) or mean (SD) depending on variable distributions, whereas categorical variables will be summarized by the number and percentage of events. Chi-square tests and Mann-Whitney methods will also be used for comparative purposes. All analyses will be performed both on intention to treat and as treated populations.

## Discussion

### Trial status

The trial is now actively recruiting at centers in the United Kingdom, France and Germany.

Through investigator meetings and feedback, a few recurrent operational issues have arisen. Many centers have an admission for completion of all TAVI-related tests prior to a multidiscliplinary meeting decision at a later date. This then requires a further attendance to hospital for consent, baseline assessments and randomization. This can be expedited if a Heart Team can convene for a decision while the patient is an inpatient so that they can be approached, consented, *etcetera.* Regarding the consent process, this target population is often rather elderly and may, quite appropriately, be apprehensive regarding the TAVI procedure. So investigators must carefully and simply explain the trial and the consent process. Care must also be taken in assessment of the level of angina given its importance in the inclusion/exclusion criteria.

We are optimistic that the trial has achievable enrollment targets and will answer an important question in this nascent field.

## Abbreviations

AKIN: Acute kidney injury network
AS: Aortic stenosis
BMS: Bare metal stent
CABG: Coronary artery bypass grafting
CAD: Coronary artery disease
Cx: Circumflex artery
DES: Drug eluting stent
DSE: Dobutamine stress echocardiography
LAD: Left anterior descending artery
LMS: Left main stem
LV: Left ventricular
LVEF: Left ventricular ejection fraction
MACCE: Major adverse cardiovascular or cerebrovascular event
MDT: Multidisciplinary team
MI: Myocardial infarction
PCI: Percutaneous coronary intervention
RCA: Right coronary artery
RCT: Randomized controlled trial
sAVR: Surgical aortic valve replacement
SVG: Saphenous vein graft
TAVI: Transcatheter aortic valve implantation
TIA: Transient ischemic attack
TTE: Transthoracic echocardiography
TOE: Transesophageal echocardiography
VARC: Valve academic research consortium
VARC2: Valve academic research consortium second guidelines.
